# Disability-Based Microaggressions, Social Support, and Psychological Distress in Aging Adults with Disabilities

**DOI:** 10.1093/geroni/igaf122.2452

**Published:** 2025-12-31

**Authors:** Dylan Serpas, Daniel Ignacio, Barbara Cherry

**Affiliations:** University of South Florida, Tampa, Florida, United States; University of California, Los Angeles, Los Angeles, California, United States; California State University, Fullerton, Fullerton, California, United States

## Abstract

**Introduction:**

Disability-based microaggressions — subtle, often unintentional discriminatory remarks or behaviors — are related to increased psychological distress among aging adults. These experiences may reinforce negative stereotypes, undermine autonomy, and contribute to feelings of exclusion. Social support is an empirically-supported moderator of the effects of discrimination, yet research examining social support and lifetime frequency of disability-based microaggressions among aging adults with disabilities (AWD) is limited. This study explored whether social support moderates the relationship between lifetime experiences of disability-based microaggressions and psychological distress.

**Method:**

A national sample of 243 aging adults with disabilities aged 40 to 70 (M = 49.13, SD = 6.28; 52.7% female; 52.3% non-Hispanic white; 39.1% heterosexual; 95.5% married; 57.6% psychiatric disability) completed the Ableist Microaggression Scale, the Multidimensional Scale of Perceived Social Support, and the Depression Anxiety and Stress Scale-21 to assess depressive and anxiety symptoms. Moderated linear regression models tested whether social support moderated the association between microaggressions and distress.

**Results:**

After controlling for relevant sociodemographic and disability characteristics, disability-based microaggressions were positively associated with anxiety symptoms (b = 0.71, p=.02), while social support was not (b=-0.02,p=.366). Microaggressions were not significantly correlated with depressive symptoms (b = 0.71,p=.02) when accounting for social support (b=-0.05,p=.040). No moderation effects of social support were observed.

**Discussion:**

Findings suggest disability-based microaggressions are related to anxiety symptoms, but social support did not attenuate this relationship. Results highlight the need for targeted interventions to reduce the burden of microaggressions and promote inclusive environments for aging adults with disabilities.

